# Smart home environment data across 4 European countries

**DOI:** 10.1016/j.dib.2025.111636

**Published:** 2025-05-09

**Authors:** Stefan Winterberger, Dmitriy An, Martin Biallas, Andrew Paice

**Affiliations:** Lucerne University of Applied Sciences and Arts, Engineering and Architecture, iHomeLab, Horw 6048, Switzerland

**Keywords:** Ambient assisted living, Active assisted living, AAL dataset

## Abstract

This paper describes a dataset of anonymised smart home environment data that was collected during a project over 359 days (16.05.2023-08.05.2024). The dataset contains information about temperature (°C), humidity (%), ambient light (lux), CO_2_ (ppm), VOC (ppm), sound pressure level (dB) in a time interval of 2–5 min in addition to event based data from PIR-Sensors and door contact sensors. Additionally, time and location information for each data point is available in the form of a time stamp, the user ID, the room and the country. The dataset was collected in 4 different European countries from a total of 62 users at their residential settings. Different installations had different sets of sensors, meaning not all parameters were measured at every location. The target group for the field trials was elderly people 65+.

During the project it could be shown that the data can be used to estimate presence in a room, based on the environmental data only, where the output of the PIR-Sensors were used as proxy labels. The weakness of the dataset is the lack of validated ground truth, which makes supervised learning approaches difficult. The strength of the dataset lies in the variety of sensors including sound pressure and the long period (nearly 1 year) of high frequency measurements in different countries.

Collecting data in real-world residential settings is challenging, but by making this dataset publicly available, we provide researchers with a valuable resource to explore smart home applications, presence detection, and environmental monitoring in everyday life.

Specifications Table

**Table:** 

Subject	Human–Computer Interaction
Specific subject area	Dataset of smart home environmental data, collected from 62 residents of elderly people (65+) from 4 European countries during one year.
Type of data	Table data, Time series data, Raw, Filtered, CSV
Data collection	The data was collected from 3 different types of commercially available smart home sensors installed at the residential home in different rooms. The Anyware Sense is a multi sensor that measures temperature, humidity, relative loudness, ambient light, CO_2_ and VOC. This sensor was connected to the habitants WiFi Access Points and sent the collected data to the Anyware cloud. The Anyware cloud forwarded the aggregated data (approx. 5 min intervals) of the field trial participants to an MQTT Message Broker, from where it was collected for the dataset. The Anyware Sense was under development during the time of the project. The second sensor was an Aqara Motion Sensor. This is a PIR Sensor (Passive Infrared) that detects movement in a defined area, and sends the movement events via the ZigBee protocol to a ZigBee Gateway. As ZigBee Gateway, a Homey Bridge was used. The Homey Bridge forwarded the movement events to an MQTT Broker, from where it was collected for the dataset. The third sensor was a Reed Contact Sensor from the company Frient. They were placed at the main entrance of some apartments to gather additional info, whether the door is open or closed. Similar to the Aqara Sensor, this sensor sent its data to the Homey Bridge and got then forwarded to the MQTT Broker. The data of the motion and door contact sensors were forwarded upon occurrence and not aggregated.
Data source location	62 residential homes spread across Denmark, Portugal, Romania and Switzerland
Data accessibility	Repository name: Zenodo Data identification number: 10.5281/zenodo.14243470 [7] Direct URL to data: https://zenodo.org/records/14243471 Instructions for accessing these data: Download the 2 files ‘event_data.zip’ and ‘periodic_data_monthly_csv.zip’ from the zenodo direct URL above and unzip them.
Related research article	None

## 1 Value of the Data

• The dataset contains data from 4 different European countries (Denmark, Portugal, and Romania, Switzerland) from a total of 62 users. These countries were selected as partners due to their involvement of End-User Organizations that participated in the project. These organizations were chosen not only for their geographic diversity but also because they recognized the value of the project and were willing to contribute to its success. The geographic spread allows researchers to research potential regional variations in indoor environmental conditions, which can be essential for understanding how climate, culture, and infrastructure may influence indoor environments across different European climates and socio-cultural contexts.
• The dataset covers a nearly year-long period, allowing researchers to investigate seasonal trends and long-term behavioural patterns within residential environments. Such a long continuous data collection period enables more robust analyses of time-based variations, such as seasonal impacts on indoor air quality, temperature, and humidity levels, especially relevant in tracking environmental effects on health and well-being over extended periods.
• With data on temperature, humidity, light levels, CO_2_, VOC, sound pressure level, and events from PIR and door contact sensors, this dataset offers various insights into residential home environments. This diverse range of variables provides a good foundation for interdisciplinary research, enabling studies across fields such as environmental science, health, gerontology, and smart home technology.
• The integration of sound pressure level (decibels) measurements is rare in smart home datasets. This data can be valuable for research on noise pollution and could support studies focused on noise as a factor in user activity and room presence.
• Due to the inclusion of PIR sensor data, this dataset provides proxy labels for room presence estimation. While not fully labelled, the data can be instrumental in developing machine learning models for activity and presence estimation using only environmental parameters. This proxy labelling set-up encourages innovative methodological approaches for addressing similar challenges in other datasets.
• Collected from a target group of elderly users aged 65+, the dataset is valuable for ageing-in-place studies and smart home technologies designed for older adults. Researchers focused on improving quality of life and independent living solutions for the elderly can use this data to explore how environmental factors impact their daily lives, health, and comfort in residential settings.

## 2 Background

During our international project, funded by the European AAL Joint program, a significant amount of resources were used to collect data to use within the project. A goal of the project „AAL 4 All“ (A4A) was, to develop a platform that can handle sensor inputs from various sensors and combine them, so a potential increase in value could be derived compared to watching each individual sensor alone [8]. (11.03.25) As [9] (Münzner et al., 2017) could show, modern sensor fusion techniques of multi-modal sensor data are superior to earlier algorithms. Further, the system should be invisible for the user. The user should not have to configure a complex set of rules.

The collected dataset could be used within the project, to develop a machine learning model, which aims to detect presence in a room, only based on environmental data that is collected commonly in a typical smart home [10] (Kang et al., 2017).

## 3 Data Description

### 3.1 Sensor types

In the dataset, three different sensor types were used. One of the sensors in different variants.

**Anyware Sense (Multi sensor prototype)**

https://anyware.solutions/wp-content/uploads/2024/08/Anyware-Sense_Technical-Product-Sheet_EN_2024.pdf [1] (20.12.24)

• BASIC: Temperature, Humidity, Ambient Light, Sound Pressure Level
• VOC: Additional VOC Sensor
• CO_2_: Additional CO_2_ Sensor

**Aqara Motion Sensor (RTCGQ11LM) (PIR Sensor)**

https://www.aqara.com/de/product/motion-sensor-p1/ [2] (20.12.24)

**Frient door contact sensor (Reed sensor)**

https://frient.com/products/entry-sensor/ [3] (20.12.24)

For the Aqara and Frient sensor to forward their data, an additional **Homey-Bridge** was installed per user:

https://homey.app/de-ch/homey-bridge/ [4] (20.12.24)

### 3.2 Data collection period

First time stamp: 2023-05-16T10:33:34.167

Last time stamp: 2024-05-08T10:38:33.434

### 3.3 Data format

CSV File format. To reduce the file size, the data is split into multiple files. The event_data.csv contains all data of the PIR and reed sensors. The periodic data like temperature, etc. is split into one file per month of the field trials.

**Event based data (event_data.csv)**

Usage example

**Fig. 1:**
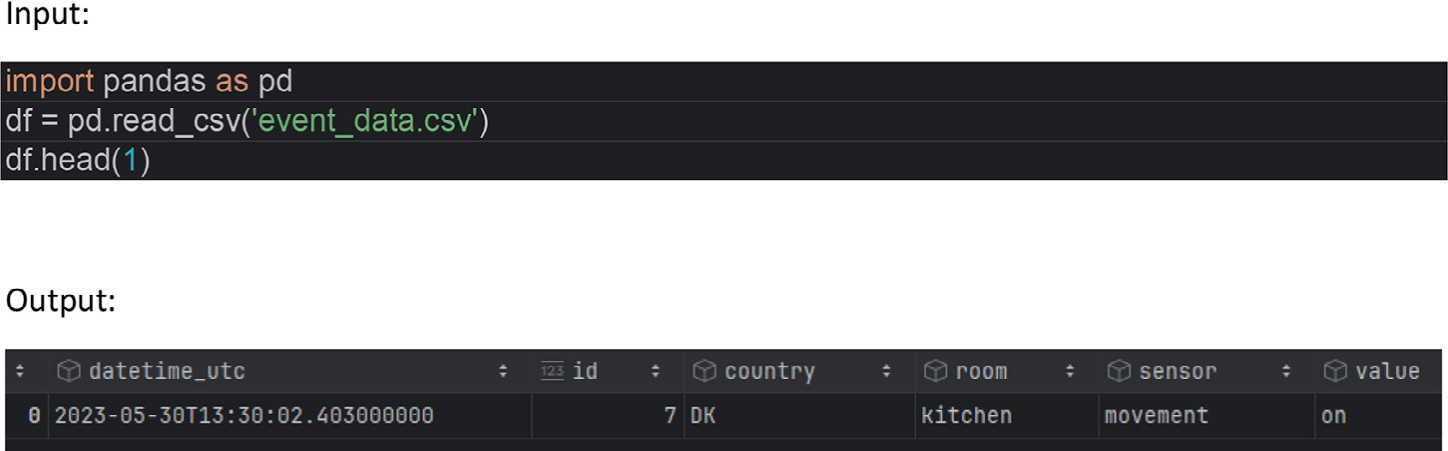
Resulting Pandas Dataframe with columns: datetime_utc, id, country, room, sensor and value (column description below).

**Fig. 2:**
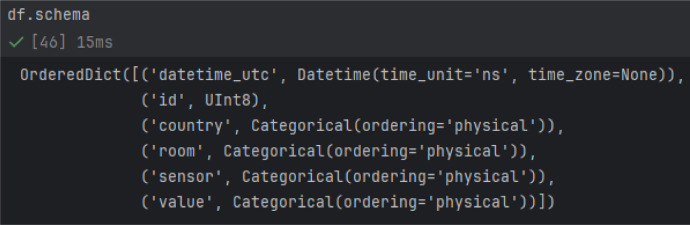
Schema of the Dataframe with according data types of the columns.

Load the data in Python 3.10 with Pandas 2.0.0 (Figs. 1 and 2)

**Periodic data (periodic_data_<year>_<month>.csv)**

**Fig. 3:**
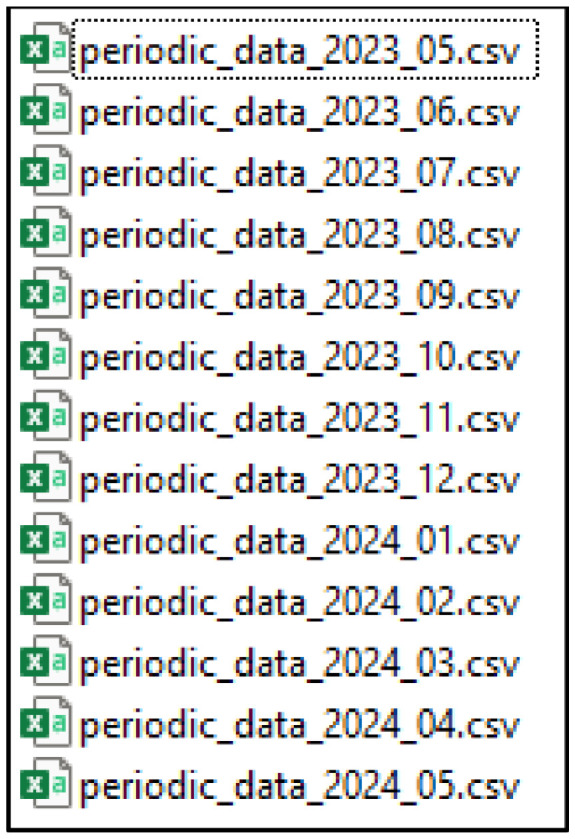
List of the CSV files with the periodic data. One file per month.

Files split by month due to file size (Fig. 3).

**Fig. 4:**
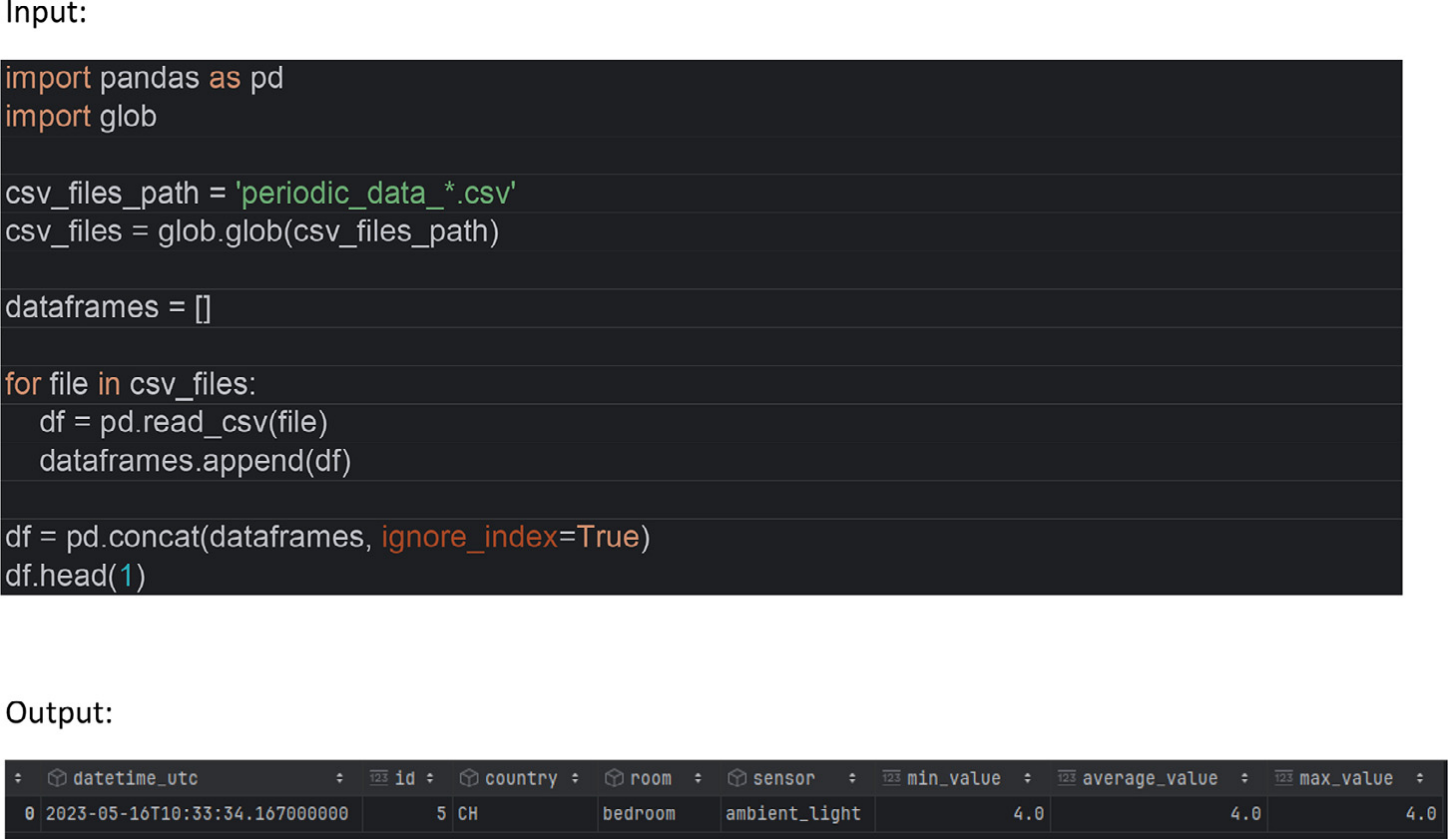
Resulting Pandas Dataframe with columns: datetime_utc, id, country, room, sensor, min_value, average_value and max_value (column description below).

Load the data in Python 3.10 with Pandas 2.0.0 all periodic data files into a single data frame with Fig. 4:

**Description of fields**

Common columns in both data frames

***datetime_utc***

Datetime as String in the format %Y-%m-%dT%H:%M:%S%.f: (Check https://docs.python.org/3/library/datetime.html#strftime-and-strptime-format-codes [6] (08.11.24) for reference). However the decimal part is in Nanoseconds. str.strptime() however expects a maximum of 6 digits. Feel free to cut the last 3 digits away like:

Input:

The timestamps are in UTC and formatted by the specification of ISO 8601. https://www.iso.org/iso-8601-date-and-time-format.html [5] (15.11.24)

***id [Int]***

An ID to map the data to the installation of a user. Values from 1 to 63 (28 is missing).

***country [String]***

Country of this User/Installation. Values:

• CH: Switzerland
• DK: Denmark
• PT: Portugal
• RO: Romania

***room [String]***

The room where the sensor of this data point was installed in. Values:

• bathroom
• bedroom
• general
• kitchen
• livingroom
• office

During the project,measurements focused on 4 rooms: Bathroom, bedroom, kitchen and living room, because these rooms were assumed to exist in most apartments. Additional rooms, that could not be assigned to one of those 4, a 5^th^ label ‚general‘ was assigned. In addition there are a few data points from office rooms.

***sensor [String]***

The type of sensor that produced this data point. Values:

• door (event based)
• movement (PIR-Sensor, event based)
• ambient_light (periodic data)
• co2 (periodic data)
• humidity (periodic data)
• sound_db_average (periodic data)
• sound_db_max (periodic data)
• temperature (periodic data)
• voc (periodic data, volatile organic components)

Columns only in event data

***value***

Value of that sensor at this event. Value differs according to sensor type:

• For ‚movement‘ sensors: The value will always be just „on“. It just indicates that this PIR Sensor triggered.
• For ‚door‘ sensors: 2 possible values: ‚open‘ or ‚closed‘.

Columns only in periodic data

To reduce traffic, the periodic data was aggregated on the backend of the multi sensors to N* minutes intervals, depending on the sensor type. Therefore, a data point contains minimum/maximum/average value of the last N minutes.

*N stands for the actual interval per sensor type. Not all data points were aggregated to the same interval. For example, temperature wasn’t expected to change that much within 5 min, whereas the interval of the sound pressure level started as well with 5 min but was reduced to 2 min later in the project.

***min_value***

The smallest value during the last N minutes.

***average_value***

The average value during the last N minutes.

***max_value***

The largest value during the last N minutes.

Meta Data

Total data points (combined event and periodic data): 74’313’246

• Periodic: 73’257’662
• Event: 1’055’584

Size of CSV Files:

• Event data (compressed / uncompressed): 6’593 KB / 58’647 KB
• Periodic data (compressed / uncompressed): 497’828 KB / 4.87 GB

Counts per month:

• May 23: 214’867
• June 23: 1’426’371
• July 23: 2’278’431
• August 23: 2’987’428
• September 23: 2’564’555
• October 23: 3’088’389
• November 23: 12’078’071
• December 23: 11’851’252
• January 24: 10’256’604
• February 24: 9’503’513
• March 24: 9’176’653
• April 24: 7’636’085
• May 24: 1’251’027

counts per sensor:

• ambient_light: 14’457’792
• co2: 3’384’878
• door: 84’991
• humidity: 14’434’119
• movement: 970’593
• sound_db_average: 11’953’602
• sound_db_max: 11’952’587
• temperature: 14’455’823
• voc: 2’618’861

counts per country:

• CH: 9’690’843
• DK: 36’359’738
• PT: 12’861’069
• RO: 15’401’596

Counts per room:

• bathroom: 17’197’279
• bedroom: 28’720’912
• general: 858’741
• kitchen: 18’801’988
• livingroom: 8’599’285
• office: 135’041

Missing incorrect data

**Minimal data clean up**

The dataset that is described in this paper was pre-processed with following steps.

***Simplified User ID***

During the project, as User Id, a unique identifier was used, which was mapped to simple values from 1 to 63 (28 is missing) in the dataset. This reduced the size of the dataset without loosing information.

***Removed ‘non-sensor’ data***

During the project, many topics from the MQTT broker were subscribed and persisted that were used for information exchange not belonging to any sensor. The data points of those topics were removed, because they bring no benefits for the data set and are not relevant after the project.

***Removed sensor data from test set ups (Non field trial users)***

There were some data points from users that were set up for testing purposes. These data points did not belong to any real residential set up and were removed.

E.g. All data points with an ‘auid’ belonging to a developer account were removed before creating the resulting data set.

**Missing event data in Switzerland**

For Switzerland (CH) there is no event data recorded. During the project set up, in Switzerland, there were only Anyware multi-sensors prototype installed without additional PIR-Sensors or door contact sensors.

## 4 Experimental Design, Materials and Methods

### 4.1 Data acquisition system

**Fig. 5:**
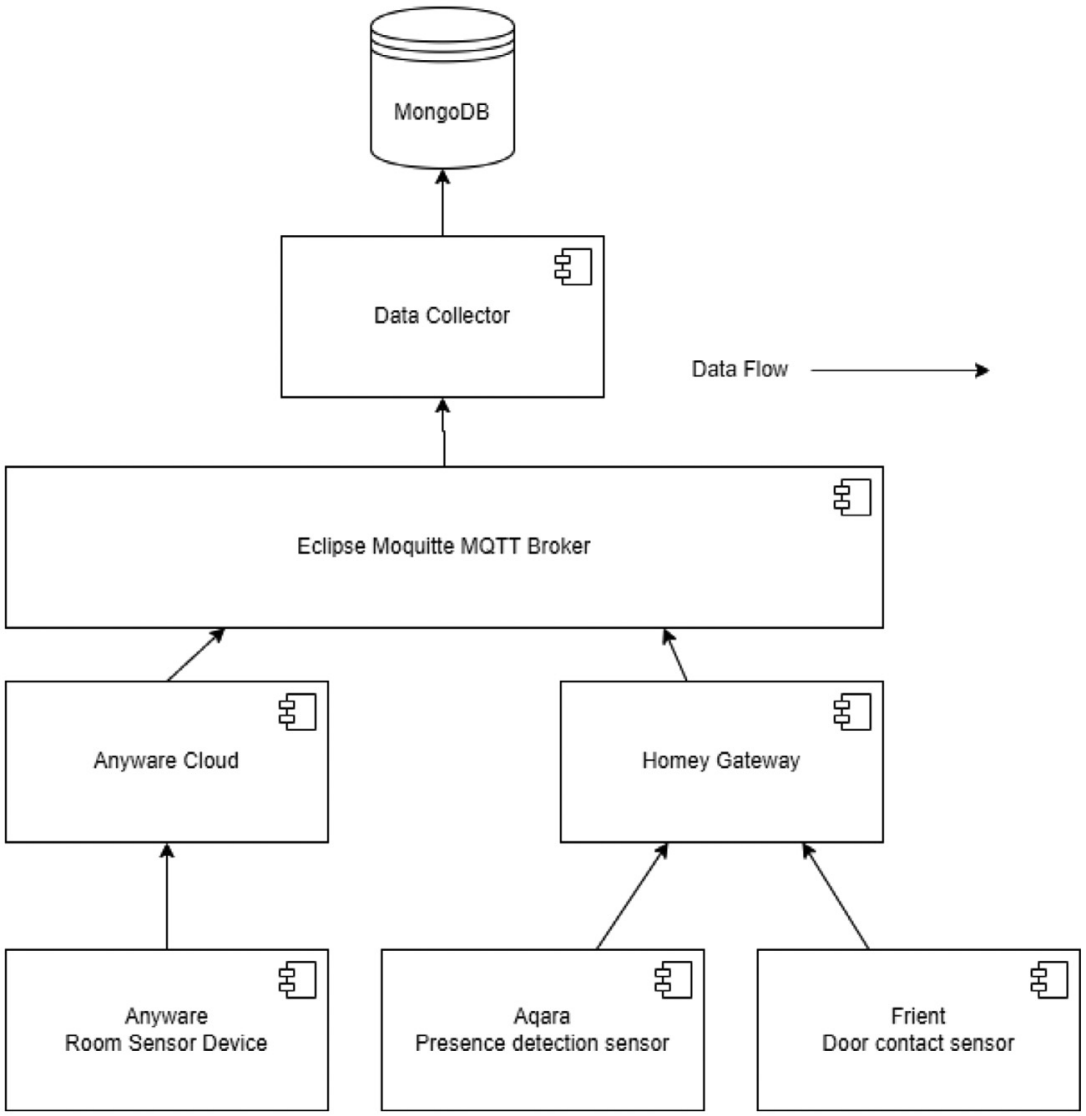
Component diagram that shows the relevant parts of the data collection pipeline.

Fig. 5 the data was collected with an MQTT Broker as common interface. A custom implemented Application subscribed to the agreed topics on the broker, and collected the data into a MongoDB. The only processing step was, to parse the user ID from the payload.

The Anyware Room Sensor Device sent their data to their own backend as usual, and the Anyware server forwarded the data of the field trial user to the MQTT Broker.

The Aqara Presence and Frient Door contact sensors sent their data via an additional Homey-Gateway directly to the MQTT Broker.

### 4.2 Calibration and validation

#### 4.2.1 Comparison of PIR data with on-site visit protocols

At the beginning of the field tests, a subset of users was selected for spot checks to verify whether the recorded data aligned with the diaries. Some of these residents received regular visits from care organizations involved in the project. These organizations were asked to log their entry and exit times for specific rooms. The recorded logs were then compared manually side-by-side with the PIR event data collected for the same users.

## Limitations

What could not be achieved during the project is a proper labelling of the data set with activities of habitants.

During the installation the project team did not follow a strong controlled approach to mount the sensors. This lead to some variability between the installations. For example, not every ambient light sensor in the living room shows daylight variations in a reliable way, etc.

The residents in the field trials lived alone. Though, because the installations were real live residential homes, the data reflects uncertainties like:

• It cannot be excluded that a small number of users might have pets, which might have triggered the PIR sensors.
• The users might have had more or less visits from relatives and/or care organisations.
• The user might have had absences for more than one day, because of hospitalisations, etc.

There are some gaps where data could not be collected properly due to server maintenance, or outage of single sensor devices or gateways.

Not all installations could be installed at day 0 of the field trials. Therefore, the dataset will show an increase in the number of data points in the beginning of the trials and a decrease in the end of the trials, where the installations were built back.

## Ethics Statement

The authors confirm that they have read and followed the ethical requirements for publication in Data in Brief. They also confirm that the current work does not involve animal experiments, or any data collected from social media platforms.

While this work does not involve direct measurements of human subjects or their physiological data, the environmental data collected in residential settings could allow for indirect inferences about the presence of residents. The field trial participants received all relevant information in an informed consent document, which they had the option to sign. Signing this document was a prerequisite for participation in the trials.

## Privacy Preservation

The informed consent contained context about what data is collected and that this will be used in an pseudonymous form. The collected data got assigned an pseudonymous user ID. Only the care organisations who where in contact with the users had a table to map the habitant to the data. The mapping tables were destroyed by the end of the project.

## CRediT Author Statement

**Stefan Winterberger:** Conceptualization, Software, Formal analysis, Data Curation, Writing – Original Draft; **Dmitriy An:** Formal analysis, Investigation, Data Curation, Writing - Review & Editing; **Martin Biallas:** Conceptualization, Validation, Funding acquisition, Project administration, Supervision, Writing – review & editing. **Andrew Paice:** Funding acquisition, Supervision, Writing – review & editing.

## Data Availability

zenodoSmart Home environment data across 4 European countries (Original data).
